# New Insight into the Impact of Effervescence on Gel Layer Microstructure and Drug Release of Effervescent Matrices Using Combined Mechanical and Imaging Characterisation Techniques

**DOI:** 10.3390/pharmaceutics14112299

**Published:** 2022-10-26

**Authors:** Pornsit Chaiya, Catleya Rojviriya, Wiwat Pichayakorn, Thawatchai Phaechamud

**Affiliations:** 1 Programme of Pharmaceutical Engineering, Faculty of Pharmacy, Silpakorn University, Nakhon Pathom 73000, Thailand; 2 School of Pharmacy, Walailak University, Nakhon Si Thammarat 80160, Thailand; 3 Synchrotron Light Research Institute (Public Organization), Nakhon Ratchasima 30000, Thailand; 4 Department of Pharmaceutical Technology, Faculty of Pharmaceutical Sciences, Prince of Songkla University, Songkhla 90110, Thailand; 5 Department of Industrial Pharmacy, Faculty of Pharmacy, Silpakorn University, Nakhon Pathom 73000, Thailand; 6 Natural Bioactive and Material for Health Promotion and Drug Delivery System Group (NBM Group), Faculty of Pharmacy, Silpakorn University, Nakhon Pathom 73000, Thailand

**Keywords:** effervescence, gel layer, matrices, mechanical, imaging, characterisation

## Abstract

Gel layer characteristics play a crucial role in hydrophilic hydroxypropyl methylcellulose (HPMC) matrix development. Effervescent agents have the potential to affect the gel layer microstructures. This study aimed to investigate the influence of effervescence on the microstructure of the gel layer around HPMC matrices using a combination of texture analysis and imaging techniques. The relationship with drug release profile and release mechanisms were also examined. The high amounts of effervescent agents promoted a rapid carbonation reaction, resulting in a high gel layer formation with a low gel strength through texture analysis. This finding was ascribed to the enhanced surface roughness and porosity observed under digital microscopy and microporous structure of the gel layer under scanning electron microscopy. The reconstructed three-dimensional images from synchrotron radiation X-ray tomographic microscopy notably exhibited the interconnected pores of various sizes from the carbonation reaction of effervescent and microporous networks, indicating the gel layer on the tablet surface. Notably, effervescence promoted the increase in interconnected porosities, which directly influenced the strength of the gel layer microstructure, drug release patterns and release mechanism of the effervescent matrix tablet. Therefore, combined mechanical characterisation and imaging techniques can provide new insights into the role of effervescent agents on the gel layer microstructure, and describe the relationship of drug release patterns and release mechanism of matrix tablets.

## 1. Introduction

The matrix system is a controlled-release drug dosage form fabricated to solve disadvantages of conventional dosage forms, such as reducing the frequency of dosing and side effects [1,2,3]. Hydrophilic matrix systems are, currently, most widely used to control the release rate of drugs [3]. A hydrophilic matrix is a homogeneous dispersion of drug molecules within a skeleton, in which one or several of the excipients incorporated are hydrophilic polymers that swell when in contact with water. Hydroxypropyl methylcellulose (HPMC) has been and continues to be the most frequently used hydrophilic polymer in formulations. This compound is a cellulose derivative in which the R substituents have the general structure of methoxy- (OCH_3_) and hydroxypropyl- (CH_2_OCH(OH)CH_3_) groups [4]. The hydrophilic matrices in contact with water become hydrated instead of disintegrating. This hydration, which is due to the increase in the size of polymer molecules as a consequence of solvent entry, leads to the formation of a zone, known as the gel layer, in which the polymer transitions from the crystalline state to a rubbery state. Several transport phenomena occur through this gel layer: entry of the aqueous medium, exit of the drug to outside the system, and matrix erosion. The penetration of the medium into the matrix is accompanied by the formation of a series of fronts, which later disappear during matrix dissolution [3,5,6]. The erosion or dissolution front is located on the outer surface of matrices and separates the gel layer zone from the medium. The diffusion front is located between the swelling and erosion fronts and separates the zone of the swollen glassy layer containing the drug dissolved in the medium from the gel layer, whereas the swelling front separates the dry core zone containing the undissolved solid drug from the swollen glassy zone [7]. Colombo and colleagues explained the characteristics of the gel layer with swelled polymers, which is located between the diffusion and erosion fronts and controls the release kinetics of the drug [6]. Over time, the water penetrates into the system and modifies the characteristics of the gel layer, which becomes thicker, owing to the extension of polymer chains. The thickness of this region is a crucial factor in the drug release process and is largely influenced by the drug dose and polymer viscosity [8]. The characteristics observed during matrix swelling include an increase in the gel layer thickness, a decrease in the size of the dry core, and an increase in the diameter of the matrix with time [9]. In this study, HPMC K15M was used as a matrix-forming agent in model matrix systems. To develop a matrix tablet, it is necessary to have other excipients to improve the physical properties of the powdered mixtures formed by the drug and polymer [3]. Previous findings demonstrated that the physicochemical characteristics of co-excipients can alter the release rate and mechanisms of the drug from the matrix. Jamzad et al. explained the effect of excipient types on front movement, erosion, and drug release behaviour of hydrophilic matrix tablets. Matrices containing water-soluble excipients demonstrated more pronounced swelling front movement and hence drug release relative to the matrix tablet containing water-insoluble excipients [10]. For matrices fabricated with 20% by weight of HPMC as retardant polymers, studies have been conducted to assess the effect of adding spray-dried lactose, microcrystalline cellulose (MCC) and partially pre-gelatinised maize starch as diluents at approximately 50%. The drug release rate increased in the matrix containing MCC and spray-dried lactose, whereas the release rate of the matrix with partially pre-gelatinised maize starch decreased because starch was incorporated into the HPMC gel structure, making it stronger [11]. In the present study, the effervescent agents, which included a mixture of sodium bicarbonate (NaHCO_3_) and citric acid anhydrous (CA) at the ratio of 1.3:1 by weight, were incorporated into the HPMC matrix tablet. Carbonation reaction can occur immediately upon contact with water and produce gas bubbles (carbon dioxide; CO_2_), allowing for the rapid release of the active pharmaceutical ingredient (API) [12,13]. Effervescent agents have been widely used in floating systems which can be buoyant within the stomach [14]. Effervescent agents can react with hydrochloric acid when systems come into contact with gastric fluids and CO_2_ gases are produced. The liberated CO_2_ gases are entrapped in the hydrocolloid matrix which causes tablet buoyancy and influences the drug release behaviours [15,16]. For effervescent floating systems, swellable polymers are mixed with effervescent agents such as NaHCO_3_, calcium carbonate, tartaric acid, and CA [17,18]. Effervescent floating systems can be categorised into single-layer, bilayer and multiple-unit systems which depend on different fabrication methods [14]. Jiménez-Martínez et al. reported that the addition of NaHCO_3_ into the HPMC matrix formulation could improve gastric retention time by increasing the hydration of tablet and increasing the surface area of drug diffusion [19]. Additionally, the increment of NaHCO_3_ content decreased the drug release rate from the matrix because the CO_2_ gas bubble may have obstructed the drug diffusion path [19]. In addition to preparation and formulation factors, pH variation in the gastrointestinal tract should also be considered. For instance, the administration of a dosage form together with 240 mL of water can increase the pH value up to 4.6 [20,21]. Furthermore, the acidity of various parts of the human digestive system differs significantly [21,22]. This pH variation can affect the carbonation reaction of effervescent agents and might alter the floatation and drug release properties of the system. Typically, organic acids such as CA, malic acid, fumaric acid and tartaric acid have been used in combination with NaHCO_3_ as effervescent agents. This combination might reduce the impact of pH variation on tablet buoyancy properties in the stomach [23]. This is one of the reasons why the mixture of NaHCO_3_ and CA was chosen as effervescent agents in this study. To date, effervescent floating system have been successfully developed as commercial products in various drugs. For example, fluoroquinolone antibiotics including ofloxacin (Zanocin OD^®^) and ciprofloxacin (Cifran OD^®^), anti-hyperglycemic metformin hydrochloride (Riomet OD^®^), and anti-hypertensive prazosin hydrochloride (Prazopress XL^®^) [14,24]. In addition, previous research has demonstrated that CO_2_ liberation from effervescent agents can promote porous structures in polymer membranes [25] and hydrogels [26]. This condition may affect the microstructure of matrices, resulting in altered drug release and release mechanisms of HPMC matrices. However, no previous study has reported the modification of hydrophilic HPMC matrix tablets using effervescent agents and their effect on the microstructure of the matrix system.

Currently, a variety of imaging techniques are used within the pharmaceutical field to better understand and characterise a wide range of phenomena associated with drug release from hydrophilic matrix tablets. These imaging techniques include near infrared (NIR), magnetic resonance imaging (MRI), nuclear magnetic resonance (NMR), terahertz, confocal laser scanning microscopy (CLSM), ultraviolet (UV) dissolution imaging and synchrotron radiation X-ray tomographic microscopy (SRXTM). NIR has been used to monitor the development of the gel layer formed in compacts containing HPMC [27] and monitoring of drug release as a result of erosion [28]. MRI has been implemented to monitor the movement of water in pastes [29], model polymer dissolution [30] and structural evolution of hypromellose tablets [31]. A novel imaging application using NMR was also developed and used to monitor water front penetration [32], water mobility and drug diffusion in hydrophilic matrices [33]. Terahertz pulsed imaging has been used to monitor the swelling and drug diffusion of tablets containing HPMC [34,35]. CLSM has also been utilised in a variety of pharmaceutical applications, such as monitoring of gel layer growth and water penetration in hydrophilic matrices [36,37]. UV dissolution imaging has begun to diversify into an analytical tool for the monitoring of dissolution events. These dissolution events can be events with API [38,39], release from transdermal patches [40], dissolution behaviour of polymers [41], and swelling behaviour of the matrix tablet containing HPMC as a model hydrophilic matrix former [42]. Recently, the use of SRXTM has demonstrated a major advantage in the investigation of internal three-dimensional (3D) structures for a variety of solid objects, offering great possibilities for some quantitative evaluations and designs of solid dosage forms. Yin et al. confirmed that the interior porous channels and irregular surfaces in tablets can be quantified by fractal dimensions, which also correlated well with the drug release kinetics of felodipine osmotic pump tablets [43]. In addition, hydration dynamics and relative importance of the erosion and swelling layer were investigated using SRXTM and correlated with the felodipine release by a statistical model [44].

A texture analyser is primarily designed for the measurement of mechanical properties of products, particularly food products. Currently, it is a versatile research and development instrument that has been widely used in the food industry. By using different probes and assessment criteria, food scientists can monitor or demonstrate the texture quality and palpability of different foods [45]. The usage of texture analysers can be applied to various dosage forms. They have been used to optimise the adhesiveness and cohesiveness of water-in-oil emulsions [46], disintegration behaviour of fast-dissolving preparations [47] and evaluation of the mucoadhesive properties of various polymers [48]. In addition, texture analysers can be employed to investigate the swelling and erosion behaviour of matrix tablets in correlation with their drug liberation [49]. Moreover, some researchers investigated the thickness and maintenance of the gel layer of the matrix tablet and polymer hydration using a texture analyser [50,51,52]. The texture analyser procedure is relatively simple, versatile, and cost effective; the same instrument can possibly be used for multiple measurements by changing either the testing probes or measurement parameters.

This study aimed to investigate microstructural changes in the gel layer surrounding HPMC matrices containing effervescent agents using a combination of a texture analyser and imaging techniques, including digital microscopy, scanning electron microscopy (SEM) and SRXTM. The eutectic mixture of ibuprofen (IBU) and poloxamer 407 (P407) was employed as a model drug in matrix systems. To better understand the function of effervescent agents in HPMC matrices, the influence of effervescent agents on the microstructural changes of matrix tablets was investigated and correlated these parameters with drug release profiles and mechanisms. To observe the influence of medium types on the behaviour of the HPMC matrix, hydrophilic HPMC matrix tablets with various amounts of effervescent agents were also studied in pH-changed media (pH 1.2 HCl buffer (0.1 N) and phosphate buffer pH 6.8).

## 2. Materials and Methods

### 2.1. Materials

IBU (Lot No. 4000/1101/18/A-0150B), which was purchased from PC Drug Co., Ltd., Bangkok, Thailand, was used as the model drug, whereas P407 (lot no. WPDF563B, BASF, Ludwigshafen, Germany) was used as the co-eutectic forming agent for IBU. The diluent in this formulation was MCC (PH101 grade; lot no. C1910113, Mingtai Chemical Co., Ltd., Taoyuan City, Taiwan). NaHCO_3_ (Batch No. AF310196, Ajax Finchem, Seven Hills, NSW, Australia) and CA (lot no. 90900209, Maxway Co., Ltd., Bangkok, Thailand) were employed as effervescent agents, and HPMC (K15M grade; Lot No. PH26012N31, DuPont (Thailand) Co., Ltd., Bangkok, Thailand) was used as the matrix-forming agent.

### 2.2. Preparation of IBU-P407 Effervescent Matrix Tablets

In this study, effervescent matrix tablets (5E and 10E) and a control tablet (IPM) were fabricated by the melt granulation method. First, IBU and P407 at the ratio of 1:1.5 by weight were co-grinded using a mortar and pestle for 10 min to induce the eutectic formation. Then, effervescent agents (NaHCO_3_ and CA at the ratio of 1.3:1 by weight), MCC PH101 and HPMC K15M were sieved through a no. 20 sieve (850 µm) and mixed with the IBU-P407 eutectic mixture for 5 min. For the control tablet, only MCC PH101 was added and mixed with the IBU-P407 eutectic mixture. The mixture was then transferred to a glass beaker and placed in a circulating water bath (model: CWB-13L, Han Yang Scientific Equipment Co., Ltd., Seoul, Korea). The mixture was stirred continuously, and the temperature was controlled at 70 °C throughout the experiment until melted granules were obtained. After 2 h of cooling down to room temperature, the melted granules were sieved through a no. 18 stainless steel sieve (1.0 mm of open diameter). Granules were compacted into 1000 mg matrix tablets using 12.7 mm round, flat and plain punches by a hydraulic press (Carver press, Wabash, IA, USA), at a compression force of 2 tons and dwell time of 10 s. Table 1 shows the compositions of the effervescent matrix tablets.

### 2.3. Evaluations of Effervescent Matrix Tablets

#### 2.3.1. Determination of Physical Properties

The thickness, hardness, diameter, and weight of effervescent matrix tablets were evaluated for their physical characteristics. Ten tablets of each formulation were tested for their diameter and hardness with a hardness tester (ERWEKA, model: TBH-325, Langen, Germany), and the thickness was measured with a thickness tester (Teclock, model: SM-112, Nagano, Japan). Tablet weights (*n* = 10) were determined using an analytical balance (model: CP224S, Sartorius, Göttingen, Germany), and the average weight and standard deviation (SD) were calculated. The statistical significance of the obtained data was examined using the independent t-test with a significant level at *p* < 0.05. The analysis was performed using SPSS for Windows (Version 11.5, SPSS Inc., Chicago, IL, USA).

#### 2.3.2. Determination of Gel Strength upon Hydration

**Figure 1:**
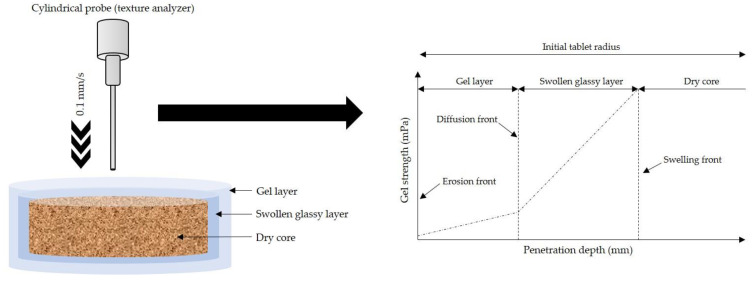
Schematic representation of gel strength determination upon hydration.

Each tablet of the effervescent matrix was placed in a dissolution apparatus filled with 900 mL pH-changed dissolution medium (including pH 1.2 HCl buffer (0.1 N) and phosphate buffer pH 6.8) and stirred with a paddle at a rotational speed of 50 rpm. This investigation was conducted in pH 1.2 HCl buffer (0.1 N) for 1.5 h. Then, the pH of the medium was raised to 6.8 until the operation was completed at 4 h. At 15 and 30 min and 2 and 4 h, five swollen tablets were carefully removed, and the gel strength was separately measured using a texture analyser (TA.XT plus, Stable Micro Systems Ltd., Surrey, UK) equipped with a 2 mm diameter flat-tipped, round steel probe (Figure 1). The test conditions were controlled at 0.2 mm/s pre-test speed, 0.1 g trigger force and 0.1 mm/s test speed. Gel strength was calculated as the ratio between the force and penetration depth of the probe inside the gel in accordance with the following equation:

(1) $$G=\frac{F}{x}\times \frac{1}{r_{p}}\times 0.0098$$

where *G* = gel strength (mPa), *F* = force (g) registered at the probe penetration, *x* = penetration depth (mm) and *r_p_* = radius of the probe (1 mm). The gel strength at the gel–solution interface was considered as the first point after the probe came into full contact with the gel (trigger force reached), and the initial noise disappeared. The average gel strength was calculated as the mean of five consequent data [53].

#### 2.3.3. Sample Pretreatment for Topological and Microtomographic Studies

According to previous reports, the freeze-drying method can maintain the microstructure of the hydration layer as much as possible with its rapid cooling process [44,54]. Therefore, a standard dissolution test was carried out to evaluate temporal changes in the microstructure of the effervescent matrix tablets during hydration. The swollen tablets were carefully removed at predetermined time intervals (0, 15 and 30 min; 2 and 4 h) together with 2 mL medium and placed individually in a glass Petri dish with a diameter of 5 cm. The glass Petri dishes containing the tablets in various states of hydration and erosion were then immediately placed in an ultra-low temperature refrigerator (model: UF-V 500, BINDER GmbH, Tuttlingen, Germany) at −80 °C for 12 h. After this process, the tablets were freeze-dried over a period of 24 h at −40 °C and 15 mbar vacuum conditions using a freeze dryer (Triad^TM^, Labcondo, Kansas City, MO, USA). The tablets were then kept in a desiccator under ambient temperature for further testing.

#### 2.3.4. Matrix Morphological Change under Digital Microscopy and SEM

After the freeze-drying process, the morphological changes in the surface and cross-sectional effervescent matrix tablets after being hydrated in the dissolution media were recorded using a digital microscope with a magnification of 26.8× (model: AM-413ZT, Dino-Lite Pro, CHOSEN Technology Co., Ltd., Bangkok, Thailand). For the surface morphological study, SEM (Maxim 200 Camscan, Cambridge, UK) was used to observe the surface morphology of dried tablets at an accelerated voltage of 15 kV and a magnification of 250×.

#### 2.3.5. SRXTM and Porosity Determination

Microtomographic images of dried effervescent matrix tablets were acquired using an SRXTM beamline (BL1.2W: X-ray imaging and tomographic microscopy) at the Synchrotron Light Research Institute (SLRI), Nakhon Ratchasima, Thailand. The synchrotron X-ray radiation was generated from 2.2-Tesla multipole wiggler at the Siam Photon Source operated at 1.2 GV. Using a filtered polychromatic X-ray beam with a distance of 32 m from the source to a sample, the experiments were conducted at a mean energy of 11.5 kV. Based on the formulations composed of IBU-P407 eutectic mixture, which can melt at low temperatures, an excessively prolonged exposure time to an X-ray beam during testing can melt the dried effervescent matrix tablet. To reduce exposure time to an X-ray beam during testing, a dried effervescent matrix tablet was divided into eight parts, and one-eighth was mounted on the stage. Then, X-ray radiographs were collected from 0° to 180° with an angular increment of 0.3°. The collected X-ray radiographs were then analysed using Octopus Reconstruction software (TESCAN, Gent, Belgium) [55] to create reconstruction images. A total of 600 reconstruction images were selected, and the porosity based on binary images was calculated using Octopus Analysis software (TESCAN, Gent, Belgium). The reconstruction images were computed using Drishti software (National Computational Infrastructure, Canberra, Australia) [56] to produce the 3D tomographic volume.

#### 2.3.6. In Vitro Drug Release Study and Release Kinetics

To investigate the effect of medium types on the drug release pattern of effervescent matrices, 900 mL pH-changed medium was used in this study. The in vitro drug release in pH 1.2 HCl buffer was conducted for 1.5 h. Then, the medium pH was adjusted to 6.8 by adding 1.09 g sodium hydroxide, 3.06 g monobasic potassium phosphate and 5.02 g dibasic sodium phosphate until completion of the operation at 24 h. The in vitro drug release was studied using a dissolution apparatus (DT 820, Erweka, Langen, Germany) and the paddle method at 37 ± 0.5 °C with a rotational speed of 50 rpm which complied with the USP43 <711> dissolution procedure [57]. At predetermined intervals, each 5 mL aliquot was withdrawn from the dissolution medium and replenished with 5 mL fresh medium. The amount of dissolved drug was measured using UV-vis spectrophotometer (Cary 60 UV-vis, Agilent Technology, Santa Clara, CA, USA) at a wavelength of 220 nm. The mean cumulative drug dissolution ± SD was calculated (*n* = 3). The mechanisms of drug release were determined by fitting the drug release data with zero-order, first-order, Higuchi’s, Korsmeyer–Peppas, Hixson–Crowell, Hopfenberg, and Peppas–Sahlin equations using DDSolver software, which is a menu-driven add-in program for Microsoft Excel (Redmond, WA, USA) written for visual basic applications [58]. Moreover, mathematical analysis was performed by calculating the coefficient of determination (R^2^), Akaike information criterion (AIC) and model selection criterion (MSC) for the drug release data with appropriate modelling.

## 3. Results and Discussion

### 3.1. Physical Properties of Effervescent Matrix Tablets

**Figure 2:**
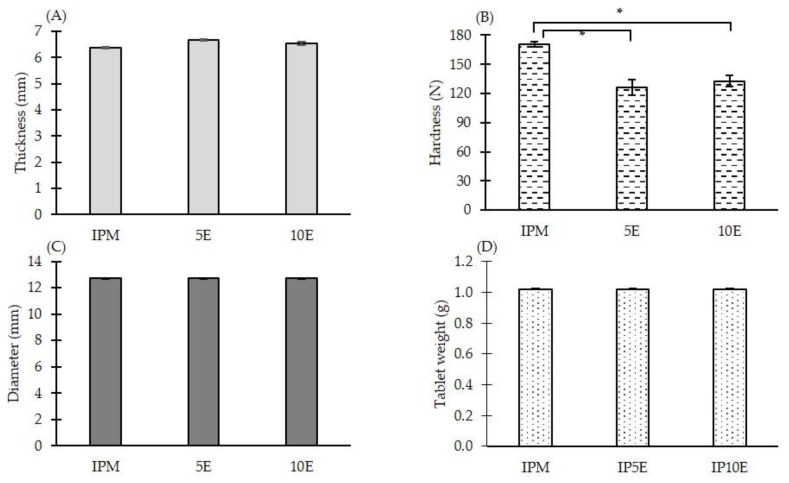
(**A**) Thickness, (**B**) hardness, (**C**) diameter and (**D**) tablet weight of IBU-P407 effervescent matrix tablets (*n* = 10) (the asterisk (*) represents a significant difference (*p* < 0.05)).

The effervescent matrix tablets were successfully fabricated by melt granulation using a circulator water bath at 70 °C. Given the effervescent matrix tablets consisting of a eutectic mixture between IBU and P407, these components can melt at approximately 50 °C [59]. This mixture acted as a meltable binder and facilitated the agglomeration of powder particles into granules. All fabricated effervescent matrix tablets had good physical appearance. The physical properties, including thickness, hardness, diameter, and tablet weight, were investigated, and are illustrated in Figure 2. No significant difference was observed in the thickness, diameter, and average tablet weight of all formulations. In the case of hardness, IPM showed the highest hardness at 170.40 ± 2.79 N. The hardness values for 5E and 10E decreased significantly to 126.20 ± 7.79 N and 132.80 ± 6.14 N, respectively, due to the incorporation of effervescent agents. Nevertheless, a non-significant difference in hardness was observed between 5E and 10E. The hygroscopic nature of effervescent agents, particularly CA, can soften tablet texture, leading to reduced tablet hardness [60,61]. The lower amount of MCC PH101 in the effervescent matrix tablets (5E and 10E) compared with the IPM tablet promoted a lower tablet hardness. MCC has an extremely low coefficient of friction and very low residual die wall pressure, which provide tablet binding properties [62,63]. MCC can also cause the adhesion between particle agglomerates, resulting in higher hardness with less friability of tablets [64].

### 3.2. Gel Strength of Effervescent Matrix Tablets after Immersion in pH-Changed Media

**Figure 3:**
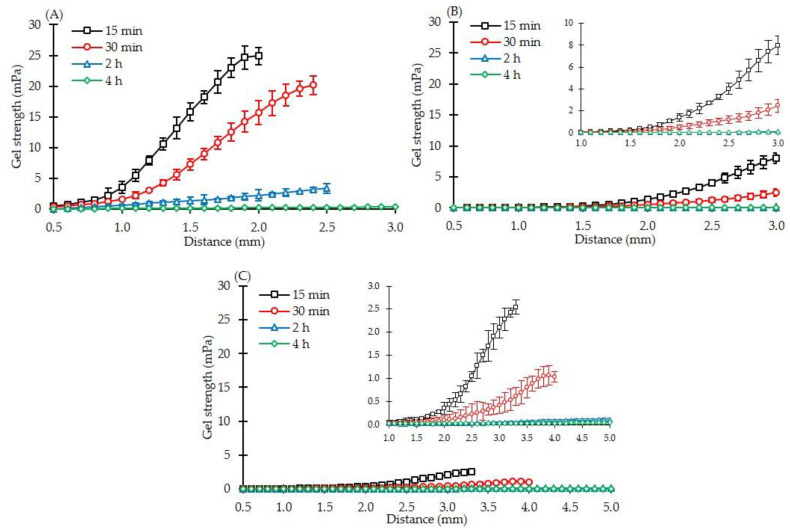
Gel strength profiles of IBU-P407 effervescent matrix tablets (*n* = 5); (**A**) IPM, (**B**) 5E and (**C**) 10E.

The effect of effervescent agents on the gel layer formation surrounding the matrix tablet has not been previously reported. In this study, the hydrated matrix tablets were characterised for their gel strength using a texture analyser at different dissolution times. Typically, three regions in a swollen matrix tablet were identified based on the degree of hydration: gel layer (highest hydration), swollen glassy layer (low hydration) and dry core (no hydration) as schematically shown in Figure 1; therefore, the mechanical properties can be identified using a texture analyser [53]. At the gel–solution boundary, the gel strength was the lowest and gradually increased toward the centre of the tablet. The swollen glassy layer (partially hydrated region) was characterised by a continuously subsided increase in gel strength until the dry core was reached (Figure 1). Figure 3 exhibits the gel strength profiles of different effervescent matrix tablets. In this study, all formulations were immersed in pH 1.2 HCl buffer (0.1 N) for 1.5 h before the pH medium was raised to 6.8 until 4 h. Thus, the sampling time points at 15 and 30 min depicted the gel strength profiles in acidic medium, and those at 2 and 4 h represented the gel strength profiles in pH 6.8 phosphate buffer. An IPM tablet consisting of a eutectic mixture between IBU and P407 and MCC PH101 was used as the control in this study. The gel strength profiles of all formulations showed the same pattern. They started with a plateau before showing a linear increase in the gel strength. When the medium pH was raised to 6.8, the gel strength profiles dramatically dropped. Typically, the low gel strength of the matrix tablets indicated a gel layer surrounding them [53]. In the case of matrix tablets containing 5% (Figure 3B) and 10% (Figure 3C) by weight of effervescent agents, in comparison with the IPM tablet (Figure 3A), the gel strength profiles of both formulations were drastically reduced. Similar patterns could be observed in both gel strength profiles. However, a slightly lower gel strength was found in higher amounts of effervescent-agent-incorporated matrix tablets. At the same level of HPMC concentration in the matrix tablets (20% by weight), the higher number of effervescent agents (10E) promoted a gel layer with a low strength (Figure 3B,C).

**Figure 4:**
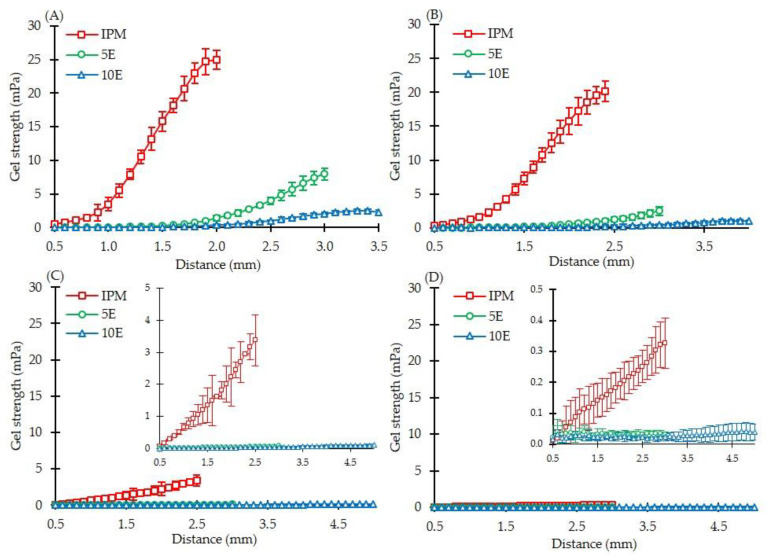
Gel strength profiles of IBU-P407 effervescent matrix tablets at different immersion times (*n* = 5); (**A**) 15 and (**B**) 30 min; (**C**) 2 and (**D**) 4 h.

As illustrated in Figure 4, the gel strength profiles of all formulations were compared at different immersion times. The effect of medium types on gel strength was also studied. In the acidic medium, the gel strengths at 15 and 30 min (Figure 4A,B) were higher than those at 2 and 4 h in pH 6.8 phosphate buffer (Figure 4C,D). This finding can be explained by the higher solubility of IBU-P407 [59,65] and gel layer-forming capability of HPMC [66,67] upon contact with the medium. There was a significant decrease in gel strength after HPMC incorporation of formulations (5E and 10E) after immersion for 30 min and 2 h. The decrease in gel strength was due to continued hydration and partial dispersion of the gel layer matrix between 30 min and 2 h. Practically, the height of the gel layer surrounding the matrix tablet can be measured by estimating the inflection point, which is the point of change in the slope of gel strength profiles. Table 2 shows the estimated gel layer height and gel strength at the gel–solution interface and the rate of gel layer formation. In pH 1.2 HCl buffer (0.1 N) for 30 min, 10E formulation showed the highest gel layer height of 2.70 ± 0.28 mm, followed by 5E and IPM tablets (1.90 ± 0.10 and 1.17 ± 0.15 mm, respectively). After the medium pH was raised to 6.8, a higher gel layer height was detected in the 5E and 10E formulations. As a result, the formation of the gel layer surrounding the tablet occurred more rapidly because of the incorporation of effervescent agents. Typically, the surface roughness increased with the increase in effervescent agents, which allowed the inward penetration of the medium, that is, deeper into the tablet, and increased the tablet wetting rate. The high wetting rate promoted the fast rate of gel layer formation in the matrix tablet [10,66,67,68]. For the gel–solution interface, the IPM tablet had the maximum gel strength of 0.213 ± 0.075 mPa at 15 min. During the immersion period, the gel strength diminished dramatically, reaching 0.041 ± 0.023 mPa at 4 h due to the dissolution enhancement of the IBU-P407 eutectic mixture. Compared with the IPM tablet, matrices containing effervescent agents (5E and 10E) exhibited similar patterns of gel strength. The gel strength of the 5E formulation was greater than that of the 10E formulation. Increasing the porosity in the formulation containing more effervescent agents can reduce the gel strength. Therefore, the gel strength profiles and gel layer heights observed by a texture analyser can be considered as crucial factors in understanding the influence of effervescent agents on gel layer formation in matrix tablets.

### 3.3. Morphological Changes Observed under Digital Microscopy and SEM

After the effervescent matrix tablets were immersed into pH-changed media, a freeze-drying approach was used in drying, and the obtained dried tablets were further investigated using imaging techniques. Although the freeze-drying method can maintain tablet microstructure in a dried state, the lack of correlation between dried state and hydrated state of matrix tablets might be found and should be recognised. However, a combination of temporal changes of tablet microstructure in the dried state using imaging techniques and mechanical properties’ characterisation in hydrated state could be of benefit to clarify the effect of effervescent agents on tablet microstructure.

**Figure 5:**
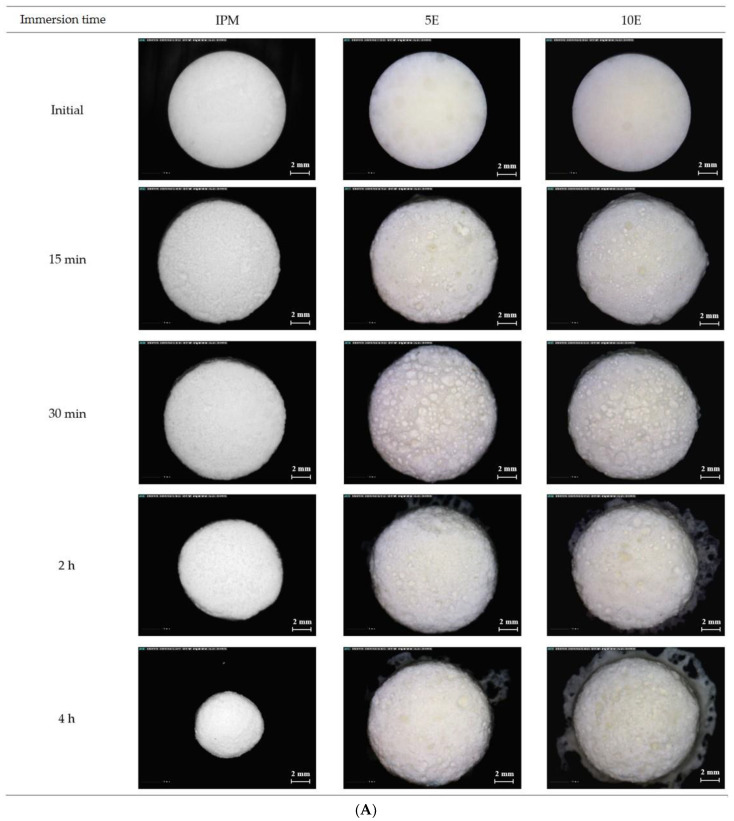

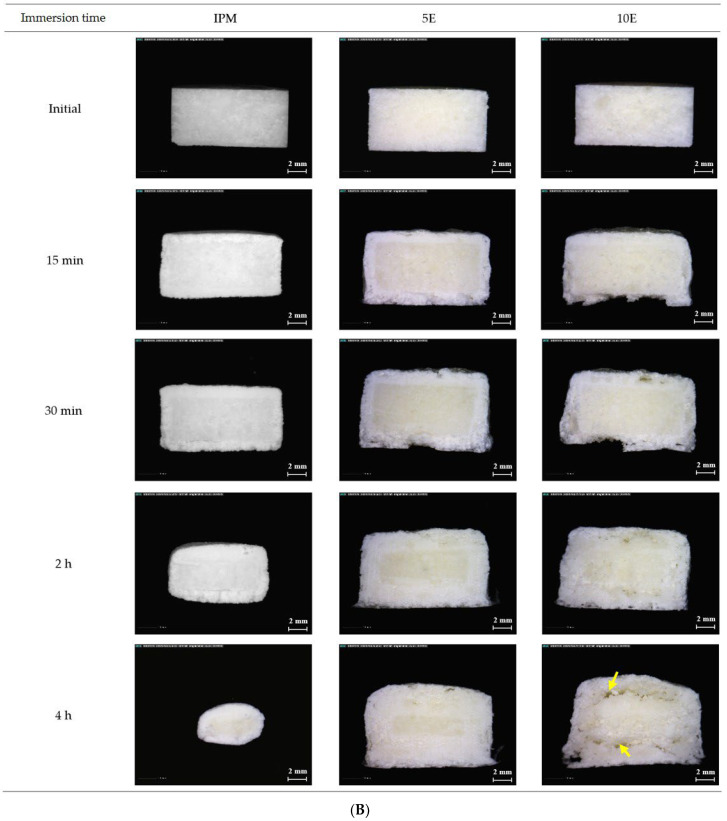
(**A**) Surface and (**B**) cross-sectional micrographs of IBU-P407 effervescent matrix tablets in pH-changed media at various immersion times (magnification: 26.8×).

Figure 5A,B show the micrographs of surface and cross-sectional effervescent matrix tablets after immersion in pH-changed media, respectively. The tablet size of the IPM formulation diminished during the immersion periods. The increased roughness observed on the IPM tablet surface after prolonged immersion in the media indicated the heterogeneous erosion on the tablet surface (Figure 5A). In the cross-sectional micrographs of IPM, a dry core was clearly identified, and was surrounded by a thin gel layer. In addition, the cylindrical shape of IPM at the initial time point altered to a tiny sphere after immersion in media for 4 h (Figure 5B). This finding confirmed the previous explanation for the gel strength profiles of IPM tablets. Dissolution improvement owing to IBU-P407 eutectic formation facilitated the erosion and decrement in tablet size as immersion time progressed [66]. Moreover, the thin gel layer surrounding the IPM tablet could not maintain the tablet shape due to its low viscosity and high solubility in the aqueous system of P407 [69]. In the case of 5E and 10E formulations, the tablet size gradually enlarged owing to the swelling and high gel layer formation of HPMC K15M in formulations. High porosity and roughness were noticeable in the 5E and 10E formulations compared with those of the IPM, but they were more pronounced in the 10E tablet (Figure 5A). The cross-sectional micrographs of 5E and 10E (Figure 5B) showed the progressively more obvious surrounding gel layer, whereas the dry core steadily diminished over time. A high porosity was observed in the 10E tablet, especially at the immersion time of 4 h. Some space was observed between the dry core and the surrounding gel layer, which did not appear in the IPM and 5E formulations (yellow arrows in Figure 5B). Moreover, the 10E tablets had the lowest gel strength of 0.006 ± 0.003 mPa at 4 h after immersion in the dissolution media. Therefore, the high concentration of effervescent agents promoted the increased carbonation reaction, resulting in a high CO_2_ gas level. Thereafter, it promoted the porosity and low strength microstructure of the matrix tablets, especially the gel layer. In addition, a large porosity can normally enhance the wetting rate and increase the rate of gel layer formation.

**Figure 6:**
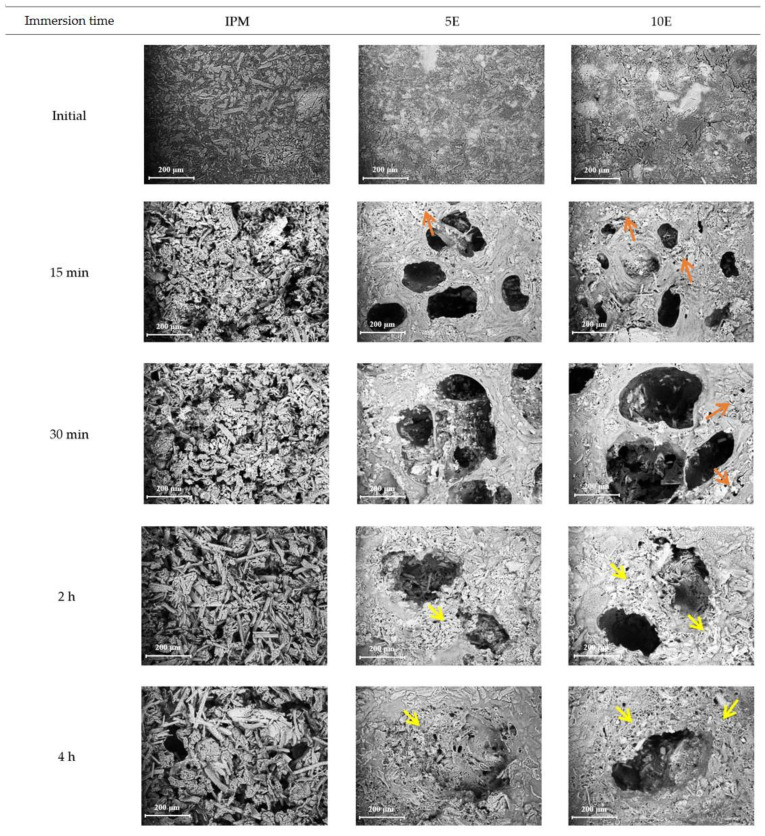
SEM micrographs of IBU-P407 effervescent matrix tablets in pH-changed media at various immersion times (magnification: 250×).

The surface morphology of all formulations was analysed by SEM (Figure 6). The SEM micrographs of 5E and 10E showed macropores in various sizes and interconnected channels distributed on the tablet surface at 15 and 30 min after immersion in the medium. These pores and channels were caused by CO_2_ generated by the carbonation reaction of effervescent excipients in the formulation, and their porosity characteristics promoted the absorption of a significant amount of water and led to swelling or gel layer formation. The gel layer was generated, as indicated by the microporous structure (brown arrows in Figure 6) [70,71,72]. The pore size was reduced, and a microporous network surrounded the pores and channels after the medium pH was elevated to 6.8 at sampling time periods of 2 and 4 h (yellow arrows in Figure 6). Moreover, the obtained pore size of the 10E tablet (diameter > 200 µm) was larger than that of the 5E tablet (diameter < 200 µm), leading to a higher rate of gel layer formation, which was evident in the microporous network of 10E tablets. This result was consistent with the above findings on gel strength and morphological changes under digital microscopy and confirmed the previous explanation about the effect of effervescence on the microstructure of matrix tablets. For IPM formulation, a tiny microporous network was unequally distributed. In addition, surface erosion was observed in the first 30 min in acidic medium, and it was more pronounced in the medium with a high pH. This finding proved the heterogeneous erosion at the surface of the IPM tablet, which was in accordance with the previous results in the gel strength study.

### 3.4. SRXTM and Porosity Determination

**Figure 7:**
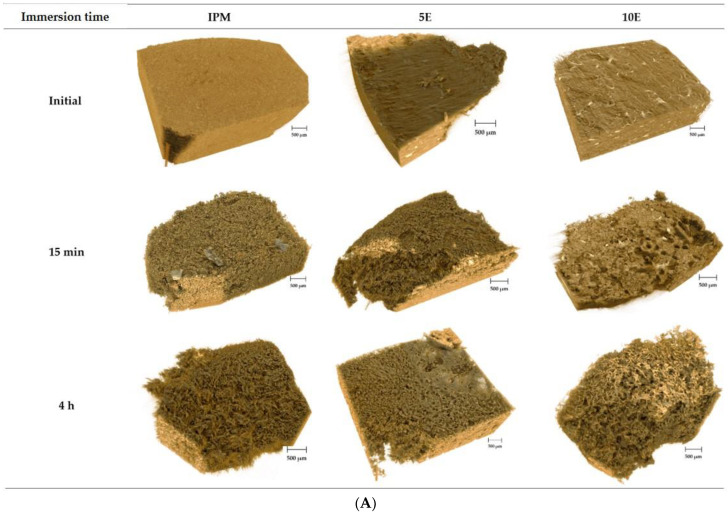

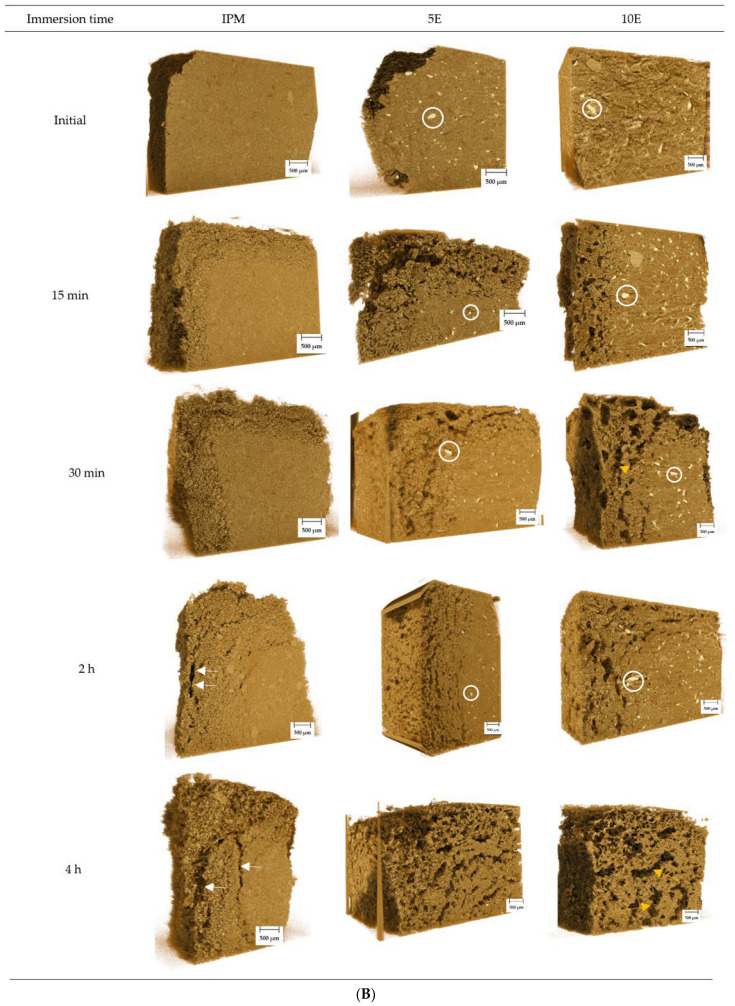
(**A**) Surface and (**B**) cross-sectional reconstructed 3D images of IBU-P407 effervescent matrix tablets in pH-changed media at various immersion times.

Figure 7A,B illustrate the surface and cross-sectional reconstructed 3D images of effervescent matrix tablets in pH-changed media at various immersion times, respectively. The 5E and 10E formulations had pores and channels on their surfaces in the first 15 min after submersion in the acidic medium. The noticeable appearance of pores and channels was observed in matrix tablets containing higher amounts of effervescent agents (10E formulation). The microporous structure was identified clearly and was inhomogeneous in terms of the size and shape of pores after immersion in nearly neutral medium at the time point of 4 h, signifying that the gel layer covered the surface of the matrices. On the other hand, surface roughness was observed in the IPM formulation at both time points in acidic and neutral media. These surface-reconstructed 3D images corresponded to the findings of the gel strength study and previously mentioned morphological changes.

As illustrated in Figure 7B, the colour gradient from light brown to dark brown indicates low to high X-ray absorption. Higher absorption referred to as higher density is mapped as white. Well-distributed, organised white spots were observed in the cross-sectional reconstructed 3D images of 5E and 10E (white circles), and they were especially greater in the 10E formulation. These white spots diminished over time and were absent in the porous structure at 4 h after immersion. These white spots, however, were not observed in the IPM tablet. The detected white spots represented bicarbonate salts in the 5E and 10E formulations, based on the X-ray absorption behaviour of the metal salt [73,74]. Given its mainly density-based contrast, SRXTM is well suited for the detection of voids or pores in the matrix, providing high-resolution images [75,76,77]. For IPM tablets, the difference in density was observed and identified in the outer and inner layers of the tablets. After the tablets were immersed in the media, the surrounding water was ingressed. Subsequently, the tablets gradually expanded and eroded, as internal cracks in the hydrate tablet formed after immersion in the media for 2 and 4 h (white arrows in Figure 7B). The water-soluble components, such as P407 and IBU-P407 eutectic mixtures in the tablets, were dissolved and then released into the medium, resulting in heterogeneous surface roughness on the surface of tablets depending on their distribution [76]. In the cases of 5E and 10E, at the initial stage (15 and 30 min), interconnected pores of various sizes and shapes were observed at the outer layer of tablets. The surrounding gel layer appeared as the pore size diminished, and the microporous structure increased steadily when formulations were immersed in a medium with a higher pH at 2 and 4 h. These characteristics were in accordance with the morphological changes observed during digital microscopy and SEM.

**Figure 8:**
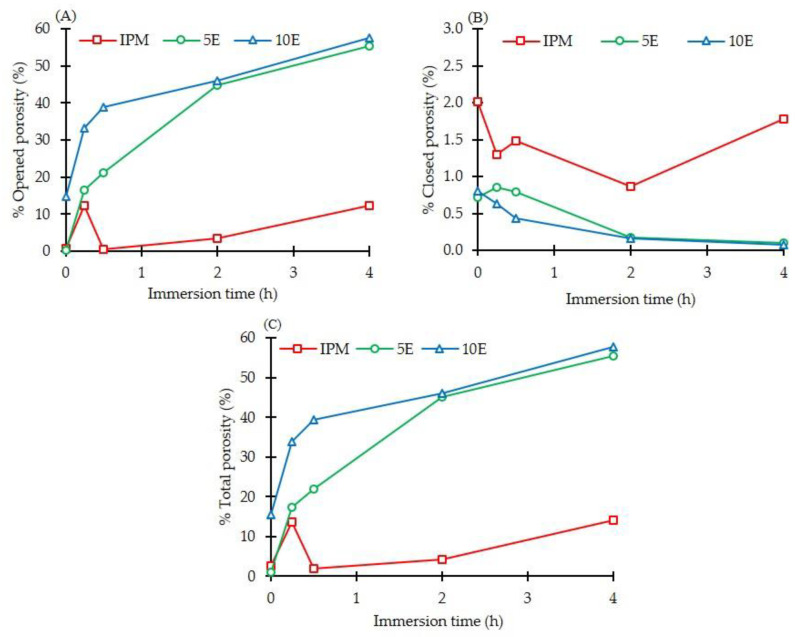
(**A**) Opened, (**B**) closed and (**C**) total porosities of IBU-P407 effervescent matrix tablets in pH-changed media at various immersion times.

The porosity parameter, which was also the important feature retrieved from SRXTM, was determined from 600 reconstruction images using Octopus Analysis software. Figure 8 illustrates the opened, closed, and total porosities of all formulations. According to the study by de Terris and colleagues, closed porosities are completely isolated inside a dense material, whereas opened porosities are pores near the part of the surface which is not completely enclosed [78]. The highest values of opened (57.62%) and total (57.69%) porosities were evident in 10E (Figure 8A,C, respectively) after immersion in dissolution media for 4 h, whereas 5E exhibited lower values of 55.42% and 55.52%, respectively (Figure 8B). Therefore, carbonation reaction promoted the interconnected pores in Figure 7B (orange arrows), and closed porosities were found in less than 1% of cases. This finding was in accordance with the study by Hesaraki et al. [79]. They developed a macroporous calcium phosphate cement matrix using a mixture of NaHCO_3_ and CA as effervescent agents. The high number of effervescent agents provoked the increase in the total porosity and interconnected pores of cement and altered its physicochemical properties, especially the compressive strength. In the case of IPM tablets, the closed porosities were more pronounced than the opened and total porosities owing to the dissolution of water-soluble components from the formulation as explained above [76]. Therefore, the effect of effervescent agents on the microstructure of matrices was well clarified using imaging techniques, including morphological study under digital microscopy, SEM, and tomographic study with SRXTM investigations. Effervescent agents promoted the interconnected pore structures or opened porosities in the microstructure of matrix tablets, particularly the surrounding gel layer. Subsequently, they affected the gel strength of matrices and the drug release behaviour of the formulation, as mentioned.

### 3.5. In Vitro Drug Release and Release Kinetics

**Figure 9:**
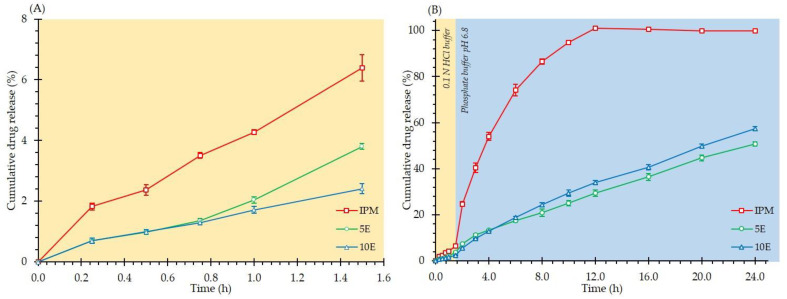
In vitro drug release of IBU-P407 effervescent matrix tablets in (**A**) pH 1.2 HCl buffer (0.1 N) and (**B**) pH-changed media.

Figure 9 illustrates the in vitro drug release profiles of effervescent matrix tablets in pH 1.2 HCl buffer (0.1 N) and pH-changed media. To determine the effect of the medium on the drug release behaviour of formulations, cumulative drug dissolution in pH 1.2 HCl buffer (0.1 N) and pH 6.8 phosphate buffer was independently fitted and displayed in Table 3 and Table 4, respectively. The best model can be derived from the highest and closest value to 1.0 for the R^2^, the highest value of MSC, and the lowest value of AIC [58]. The IPM tablets exhibited the highest drug release of 6.4% within 1.5 h in pH 1.2 HCl buffer (0.1 N). The 5E and 10E formulations showed drug releases of 3.8% and 2.4%, respectively (Figure 9A). When all formulations were further immersed in a medium with a pH of 6.8, the drug release pattern of IPM dramatically increased to around 25% after 2 h and then steadily increased to 100% within 12 h. Notably, the drug release patterns from 5E and 10E tablets continuously rose steadily, reaching 50.8% and 57.5%, respectively, at 24 h (Figure 9B).

For mathematical model fitting (as shown in Supplementary Materials), the IBU release from the IPM formulation in both media exhibited the tendencies of Peppas–Sahlin, in which the R^2^ was close to 1.0. The kinetic parameters were estimated using Peppas–Sahlin model fitting, and the release mechanism was indicated by a higher value when comparing the k_1_ and k_2_ values. A higher k_1_ value would suggest that the diffusion process was the predominant drug release mechanism, whereas a higher k_2_ would indicate that poloxamer relaxation or heterogeneous erosion was the main drug release mechanism [80]. Negative k values could typically be observed and indicated as one term in model for compensating to produce the best fit to data; nevertheless, this should not be included in interpretation of the release mechanism using the comparison of k value [80]. IPM tablets showed the highest dissolution rate of 2.7052% dissolved drug/h in pH 1.2 HCl buffer (0.1 N), with Fickian diffusion as the predominant drug release mechanism, as indicated by the higher k_1_ value shown in Table 3. When the medium pH was raised to 6.8, the positive k_2_ value (Table 4) indicated that heterogeneous erosion was the main drug release mechanism, and the dissolution rate dramatically increased to 284.2489% dissolved drug/h [80]. This finding implied that the medium pH influenced the drug release pattern of IPM tablets owing to the solubility of the IBU-P407 eutectic mixture, which was dependent on the medium pH. This finding was in accordance with the results of Dugar et al., who developed various ratios of eutectic mixture between IBU and P407 using the fusion method and discovered that the drug release pattern of this eutectic mixture could increase to around 60% within 1 h, and drug release reached 100% in pH 7.2 phosphate buffer within 10 min [59]. In the case of 5E tablets, cumulative drug release profiles in pH 1.2 HCl buffer (0.1 N) were fitted using the Hopfenberg model with the highest R^2^ value of 0.9855. Thus, heterogeneous erosion was the predominant release mechanism of 5E tablets in pH 1.2 HCl buffer (0.1 N). After the medium pH was adjusted to 6.8, Peppas–Sahlin model displayed a strong tendency with the R^2^ close to 1.0. Fickian diffusion was the main release mechanism for the drug release pattern of 5E formulation in pH 6.8 phosphate buffer, according to the higher k_1_ value in Table 4. For the 10E formulation, drug release patterns in pH 1.2 HCl buffer (0.1 N) and pH 6.8 phosphate buffer showed the best fit with the Peppas–Sahlin model, in which the R^2^ values were 0.9891 and 0.9986, respectively. However, the main release mechanism changed from Fickian diffusion in the pH 1.2 HCl buffer (0.1 N) to polymer relaxation in the pH 6.8 phosphate buffer. As a result, the medium pH possibly affected the release mechanism of effervescent matrices but slightly influenced the amount of drug that was dissolved in the dissolution medium.

In comparison with IPM tablets, effervescent agents in 5E and 10E tablets can facilitate the carbonation reaction at the beginning of immersion in the acidic medium. CO_2_ gases were generated and promoted porosity in the surrounding gel layer and tablet surface. Increased porosity caused a higher water ingression into the tablet, resulting in the rapid formation of the surrounding gel layer and retarded drug release from matrices consisting of effervescent agents. This finding can explain how 5E and 10E can retard and modulate the release of drugs from effervescent matrices. Compared with effervescent matrices in pH 1.2 HCl buffer (0.1 N), the higher concentration of effervescent agents in 10E formulation can facilitate the formation of highly porous structures. This phenomenon can promote the fast rate of water penetration, resulting in high hydration and swelling rate of HPMC K15M, which was the gel-forming agent, followed by the rapid formation of gel layer acting as a drug release barrier. This result can describe the diffusion process as the main release mechanism of 10E in the pH 1.2 HCl buffer (0.1 N) [81,82,83]. The lower concentration of effervescent agents in the 5E formulation, however, suggested that the rate of gel layer formation was slower than the rate of the carbonation reaction of effervescent agents, which resulted in a more prominent surface erosion. Thus, heterogeneous erosion was the predominant release mechanism of 5E in the pH 1.2 HCl buffer (0.1 N). After the formation of a sufficiently strong gel layer in the pH 6.8 phosphate buffer as immersion time progressed, the amount of drug released from the effervescent matrices decreased depending on the rate of drug diffusion, rate of gel layer disruption and system erosion [84,85]. This event caused the diffusion process to become the main release mechanism of 5E in pH 6.8 phosphate buffer. During drug release, erosion front occurred at the external interface between the surrounding medium and effervescent matrices [82]. Owing to the higher porosity of the gel layer in 10E, the surface area significantly increased compared with that of the 5E formulation [68], resulting in the increased surface erosion. Therefore, polymer relaxation was the predominant release mechanism of 10E in pH 6.8 phosphate buffer.

In comparison with the study of Xu et al. [86], who developed the extended-release tacrolimus matrix tablets using HPMC as a matrix-forming agent, tacrolimus was mixed with Compritol*^®^* ATO888 and Pluronic F127 to prepare a tacrolimus solid dispersion. Drug release data fitted the Kosmeyer–Peppas model very well with an n value of 0.85, indicating an anomalous transport mechanism of the tacrolimus release. The anomalous transport was a combination of diffusion and erosion-controlled release mechanisms, which was consistent with the matrix erosion and swollen behaviour of the formulation [86]. In addition, the drug release pattern can be tailored by the composition of matrix materials, such as the type and content of HPMCs and preparation methods. Viridén and colleagues investigated the effect of the chemical heterogeneity of HPMC on the release of model drug substances from hydrophilic matrix tablets. Interestingly, the interaction between the hydrophobic part of HPMC and butylparaben made the gel of tablets less hydrated and more fragile and, therefore, more affected by erosional stresses [87]. This finding revealed the possible variability in the drug release depending on the substituent heterogeneity of the HPMC used. Moreover, the effect of the solubility of the additive on the HPMC matrix tablet can be studied by a novel NMR microimaging method [88]. The rate of solvent transport into the tablet depends on the solubility of the additive and affects the release mechanism of the matrix tablets [88]. In this study, the addition of effervescent agents modified the drug-release pattern of the matrix tablets. The morphological and topological changes in the gel layer microstructures observable under digital microscopy, SEM and SRXTM, can describe the alteration of the drug-release pattern and gel strength of effervescent matrices, as described above. Therefore, combination of mechanical and imaging characterisation might be alternative methods to investigate the temporal change of tablet microstructure, and to also clarify the drug release mechanism. In addition, these combined techniques could be applied to determine the microstructure and drug release mechanism of other dosage forms such as polymer membrane for wound healing [25], hydrogels for tissue engineering applications [26] and carbohydrate-based hydrogel for substitute as articular cartilages [89].

## 4. Conclusions

Effervescent matrix tablets were successfully fabricated by melt granulation using a circulating water bath at 70 °C. The IBU-P407 eutectic mixture acted as a meltable binder, facilitated the agglomeration of particles into granules and was compressed into tablets using a hydraulic press. Based on their hygroscopic properties, effervescent agents can affect the physical properties of matrix tablets, especially tablet hardness. When effervescent matrix tablets were immersed in pH-changed media, the high amounts of effervescent agents promoted a rapid carbonation reaction, resulting in a high gel layer formation with a low strength, which was investigated by a texture analyser. This finding was described by the surface roughness and porosity observed under digital microscopy and microporous structure at the gel layer detected during SEM. However, it should be acknowledged that, from some perspectives, there is no association between the mechanical characteristics of the hydrated state and the imaging characterisation of the dried state. In addition, SRXTM was used to investigate the tablet microstructure and porosity. The reconstructed 3D images showed the interconnected pores of various sizes from the carbonation reaction of the effervescent and microporous network, which indicated the presence of the gel layer on the tablet surface. Notably, CO_2_ gases from the carbonation reaction of effervescent agents can promote the increase in opened or interconnected porosities, which affects the strength of the gel layer microstructure, drug release patterns and release mechanism of the effervescent matrix tablet. Therefore, combined mechanical characterisation and imaging techniques can provide new insights into the influence of effervescent agents on the gel layer microstructure and describe the relationship with drug release patterns and release mechanism of matrix tablets. In addition, the application of these combined techniques in different dosage forms such as polymer membranes and hydrogels would be a fascinating point for further research.

## Figures and Tables

**Table 1:** Compositions of IBU-P407 effervescent matrix tablets.

Compositions	Formulations (mg)		
	IPM	5E	10E
IBU	200.0	200.0	200.0
P407	300.0	300.0	300.0
NaHCO_3_	-	28.3	56.5
CA	-	21.7	43.5
HPMC K15M	-	200.0	200.0
MCC PH101	500.0	250.0	200.0
Total	1000.0	1000.0	1000.0

Remarks: E = % by weight of effervescent agents (the mixture of NaHCO_3_ and CA at the ratio of 1.3:1 by weight) in each formulation.

**Table 2:** Estimated gel layer height and strength at the gel–solution interface tested in pH-changed media (*n* = 5).

Immersed Time	Estimated Gel Layer Height (mm) (Mean ± SD)			Gel Strength at Gel–Solution Interface (mPa) (Mean ± SD)		
	IPM	5E	10E	IPM	5E	10E
15 min	0.93 ± 0.15	1.73 ± 0.06	2.17 ± 0.32	0.213 ± 0.075	0.034 ± 0.002	0.029 ± 0.017
30 min	1.17 ± 0.15	1.90 ± 0.10	2.70 ± 0.28	0.147 ± 0.034	0.023 ± 0.005	0.011 ± 0.004
2 h	0.60 ± 0.20	4.03 ± 0.25	4.63 ± 0.35	0.047 ± 0.022	0.015 ± 0.003	0.006 ± 0.001
4 h	0.70 ± 0.28	5.00 ± 0.00	5.00 ± 0.00	0.041 ± 0.023	0.015 ± 0.003	0.006 ± 0.003

**Table 3:** Mathematical modelling of IBU release from effervescent matrix tablets in pH 1.2 HCl buffer (0.1 N).

Modelling	Formulation	Parameters				R^2^ Adjusted	AIC	MSC
Zero-order	IPM	k_0_ = 4.3977				0.9685	0.0607	2.6787
	5E	k_0_ = 2.2858				0.9566	−3.8335	2.5255
	10E	k_0_ = 1.6920				0.9624	−11.1238	2.5833
First-order	IPM	k_1_ = 0.0452				0.9714	−0.5353	2.7781
	5E	k_1_ = 0.0232				0.9544	−3.5304	2.4749
	10E	k_1_ = 0.0171				0.9640	−11.4244	2.6334
Higuchi’s	IPM	k_H_ = 4.4344				0.9245	5.2908	1.8070
	5E	k_H_ = 2.2293				0.7662	6.3148	0.8341
	10E	k_H_ = 1.7110				0.9373	−7.2374	1.9356
Kosmeyer–Peppas	IPM	k_KP_ = 4.4822		*n* = 0.8128		0.9860	−5.1810	3.5523
	5E	k_KP_ = 2.1939		*n* = 1.2903		0.9736	−6.1534	2.9121
	10E	k_KP_ = 1.7299		*n* = 0.7760		0.9892	−20.2020	4.0963
Hixson-Crowell	IPM	k_HC_ = 0.0149				0.9705	−0.3396	2.7454
	5E	k_HC_ = 0.0077				0.9551	−3.6304	2.4916
	10E	k_HC_ = 0.0057				0.9635	−11.3240	2.6167
Hopfenberg	IPM	k_HB_ = 0.0023		*n* = 93.2625		0.9642	1.4792	2.4423
	**5E**	**k_HB_ = 0.4191**		***n* = 0.0390**		**0.9855**	**−9.8755**	**3.5325**
	10E	k_HB_ = 0.0014		*n* = 20.3999		0.9548	−9.4031	2.2965
Peppas–Sahlin	**IPM**	**k_1_ = 2.7052**	**k_2_ = 1.7390**		**m = 0.5990**	**0.9862**	**−5.4580**	**3.5985**
	5E	k_1_ = 1.2791	k_2_ = 0.8772		m = 0.9303	0.9752	−6.3063	2.9376
	**10E**	**k_1_ = 1.1047**	**k_2_ = 0.6117**		**m = 0.5863**	**0.9891**	**−20.2437**	**4.1033**

Remark: The significance of bold indicates the estimated parameters from the best-fitted mathematic model.

**Table 4:** Mathematical modelling of IBU release from effervescent matrix tablets in pH 6.8 phosphate buffer.

Modelling	Formulation	Parameters				R^2^ Adjusted	AIC	MSC
Zero-order	IPM	k_0_ = 9.8725				0.8070	50.0124	1.3762
	5E	k_0_ = 2.2892				0.9509	47.3542	2.8195
	10E	k_0_ = 2.5741				0.9692	46.1229	3.2903
First-order	IPM	k_1_ = 0.2126				0.9549	59.7612	2.9031
	5E	k_1_ = 0.0296				0.9894	31.9786	4.3571
	10E	k_1_ = 0.0345				0.9977	19.7189	5.9307
Higuchi’s	IPM	k_H_ = 25.0070				0.7664	76.2141	1.2578
	5E	k_H_ = 8.8980				0.8997	54.5326	2.1017
	10E	k_H_ = 9.9457				0.8774	60.0271	1.8998
Kosmeyer–Peppas	IPM	k_KP_ = 14.2673		*n* = 0.9289		0.9833	14.5716	3.5403
	5E	k_KP_ = 4.3409		*n* = 0.7735		0.9968	19.1651	5.6385
	10E	k_KP_ = 4.3089		*n* = 0.8179		0.9960	26.1915	5.2834
Hixson–Crowell	IPM	k_HC_ = 0.0564				0.9846	49.0139	3.9778
	5E	k_HC_ = 0.0091				0.9830	36.8165	3.8733
	10E	k_HC_ = 0.0105				0.9960	25.6593	5.3366
Hopfenberg	IPM	k_HB_ = 0.0836		*n* = 1.8288		0.9957	36.9415	5.1851
	**5E**	k_HB_ = 0.0001		*n* = 828.0660		0.9880	33.9938	4.1556
	10E	k_HB_ = 0.0027		*n* = 22.3850		0.9977	20.8258	5.8200
Peppas–Sahlin	**IPM**	k_1_ = −282.7329	**k_2_ = 284.2489**		m = 0.1229	**0.9990**	**−2.8625**	**7.8988**
	5E	**k_1_ = 3.3267**	k_2_ = 1.5962		m = 0.5233	**0.9976**	**17.8431**	**5.7707**
	**10E**	k_1_ = −12.5306	**k_2_ = 14.2621**		m = 0.2888	**0.9986**	**15.9478**	**6.3078**

Remark: The significance of bold indicates the estimated parameters from the best-fitted mathematic model.

## Data Availability

The data presented in this study are available on the request from the corresponding author.

## Supplementary Materials

The following supporting information can be downloaded at: https://www.mdpi.com/article/10.3390/pharmaceutics14112299/s1.
