# Supplementary material for: New Insight into the Impact of Effervescence on Gel Layer Microstructure and Drug Release of Effervescent Matrices Using Combined Mechanical and Imaging Characterisation Techniques
Source: Pharmaceutics. 2022 Oct 26;14(11):2299. doi: 10.3390/pharmaceutics14112299 (PMC9694726; doi:10.3390/pharmaceutics14112299)
Supplement: Supplementary file 1 [file pharmaceutics-14-02299-s001.zip › pharmaceutics-1968210-supplementary.pdf]

Supplement data for research article: New Insight into the Impact of Effervescence on Gel Layer Microstructure and Drug Release of Effervescent Matrices Using Combined Mechanical and Imaging Characterisation Techniques.

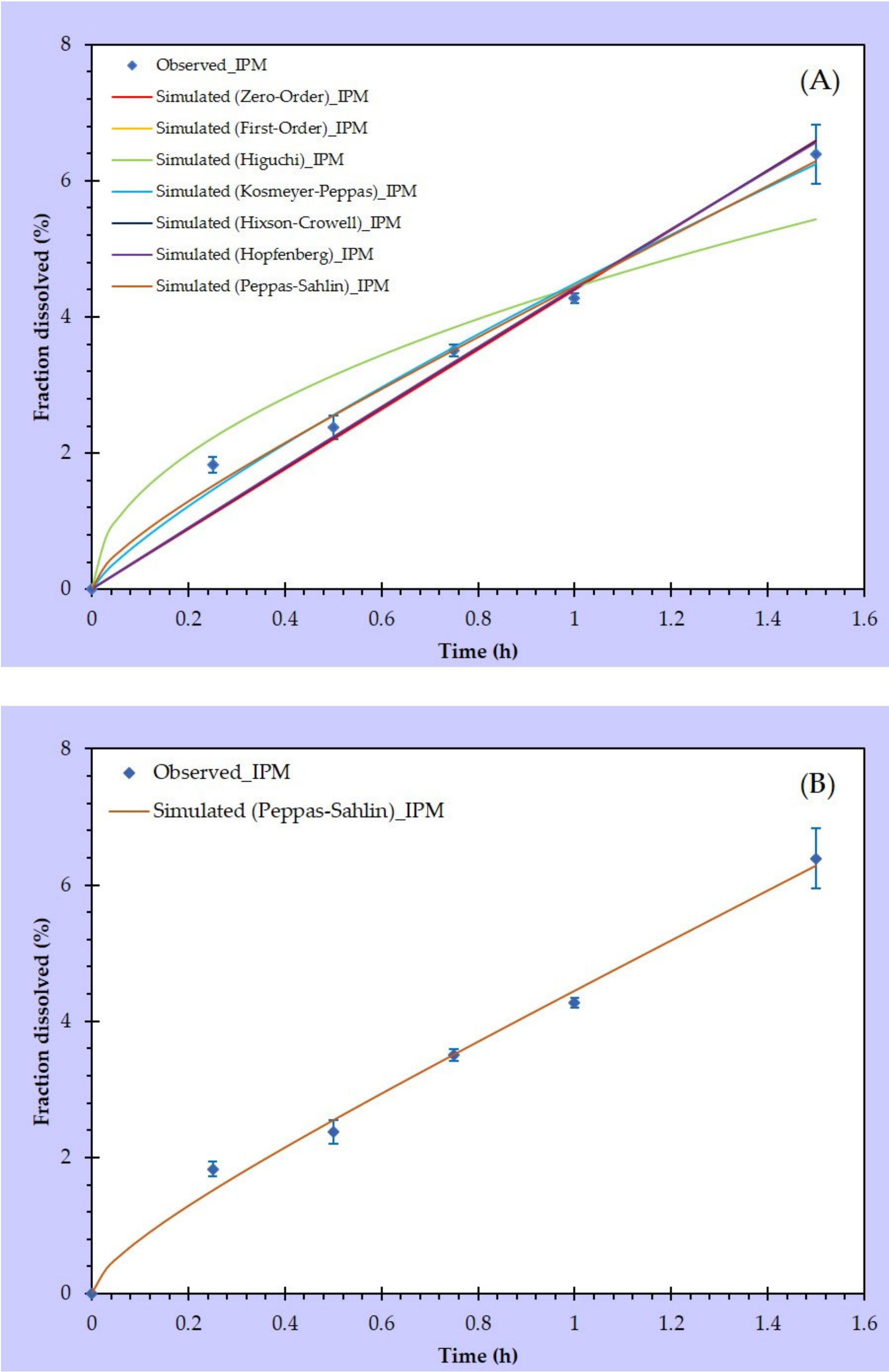

Figure S1. Mathematic model fitting of drug release profiles of IPM in 0.1 N HCl buffer: (A) All mathematic models fitting, (B) Best mathematic model fitting

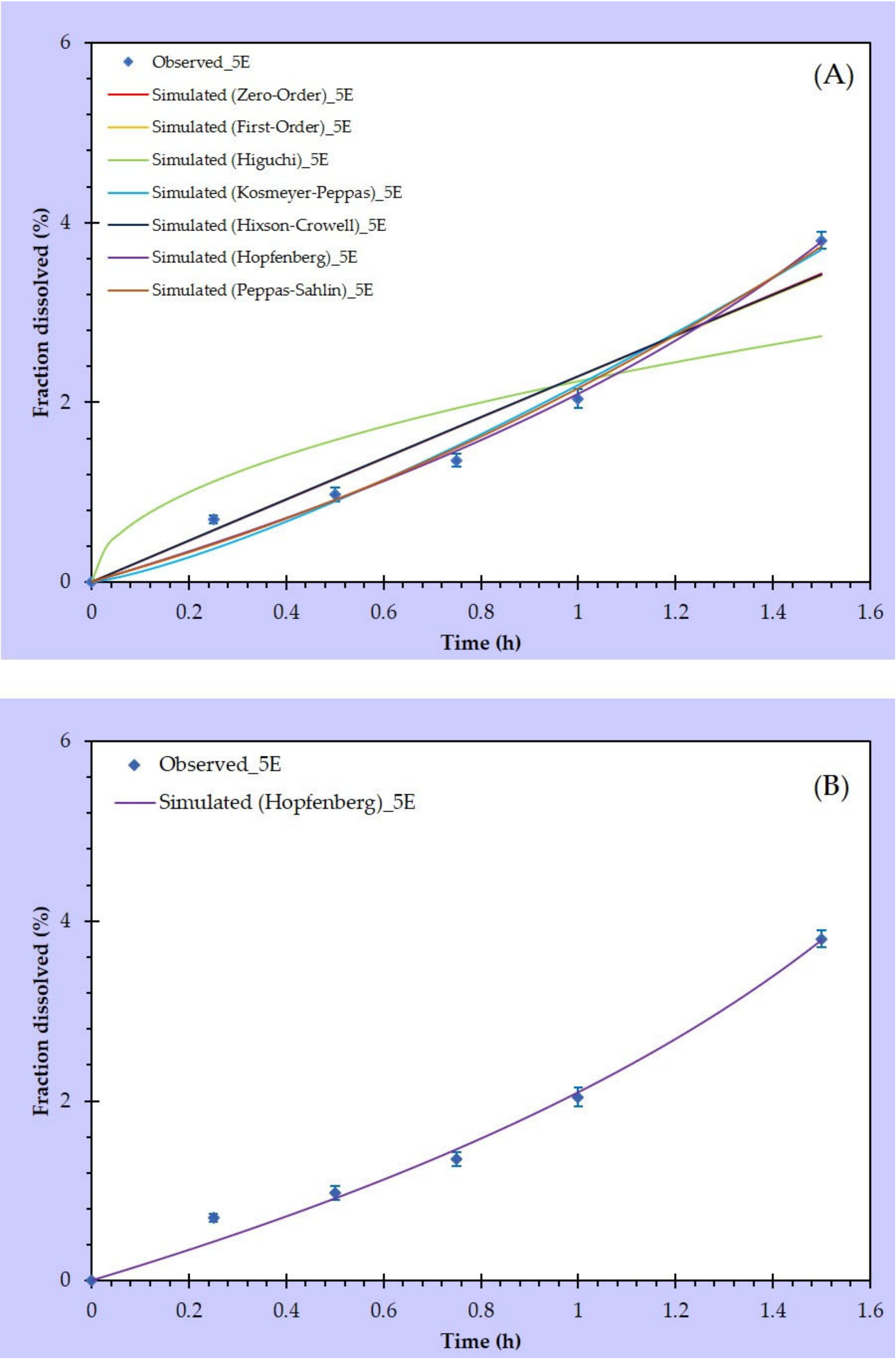

Figure S2. Mathematic model fitting of drug release profiles of 5E in 0.1 N HCl buffer: (A) All mathematic models fitting, (B) Best mathematic model fitting

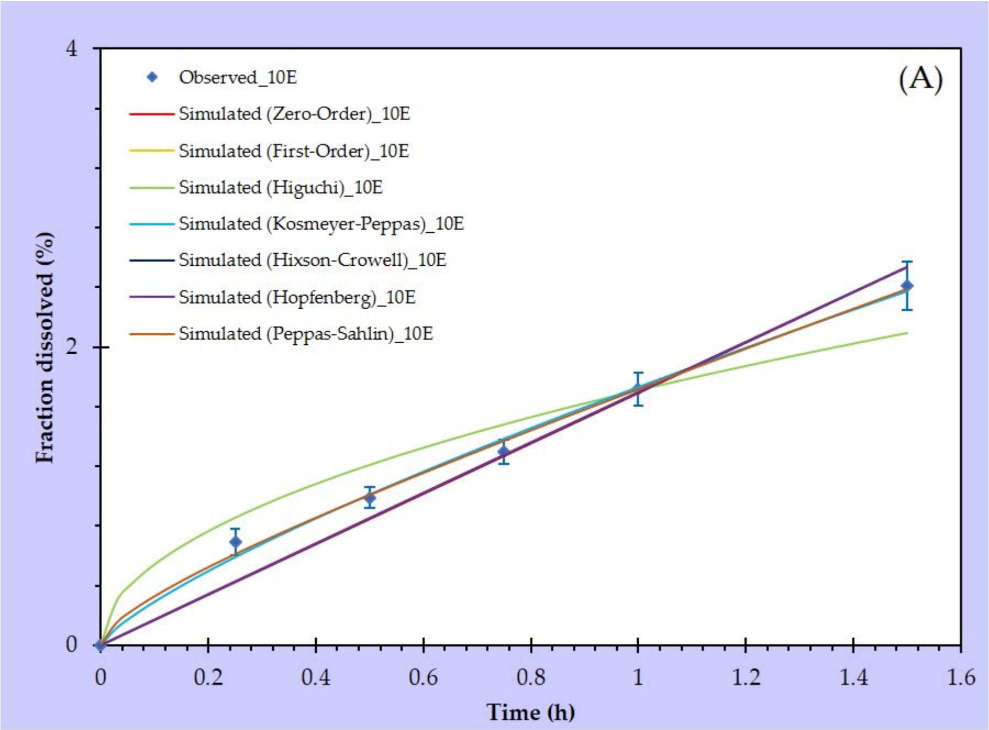

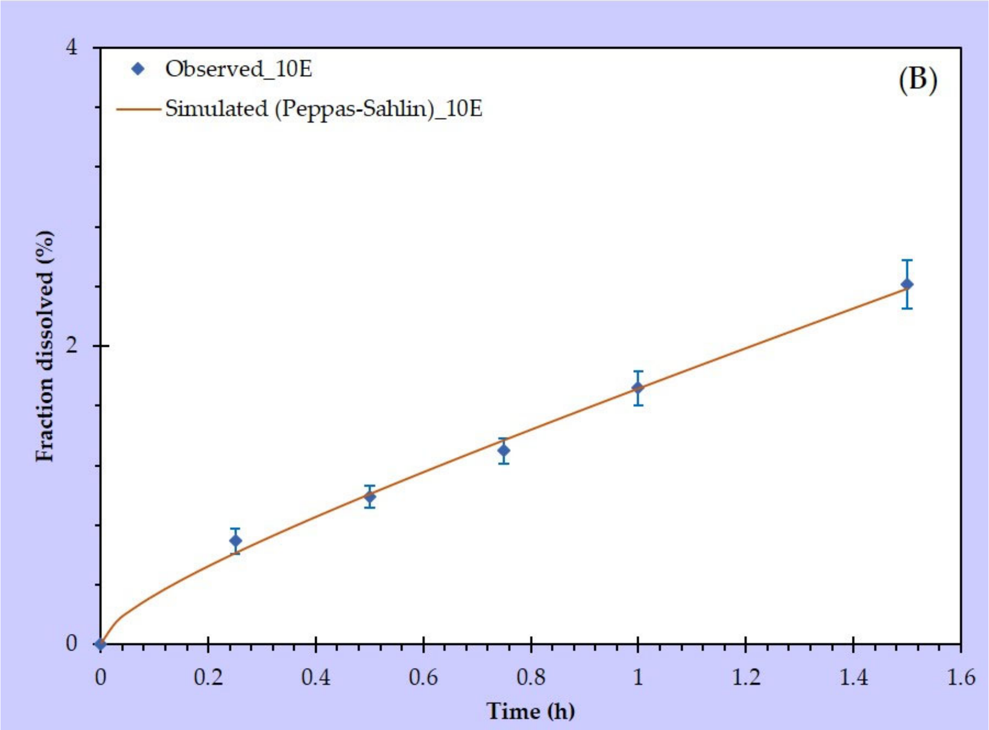

Figure S3. Mathematic model fitting of drug release profiles of 10E in 0.1 N HCl buffer: (A) All mathematic models fitting, (B) Best mathematic model fitting

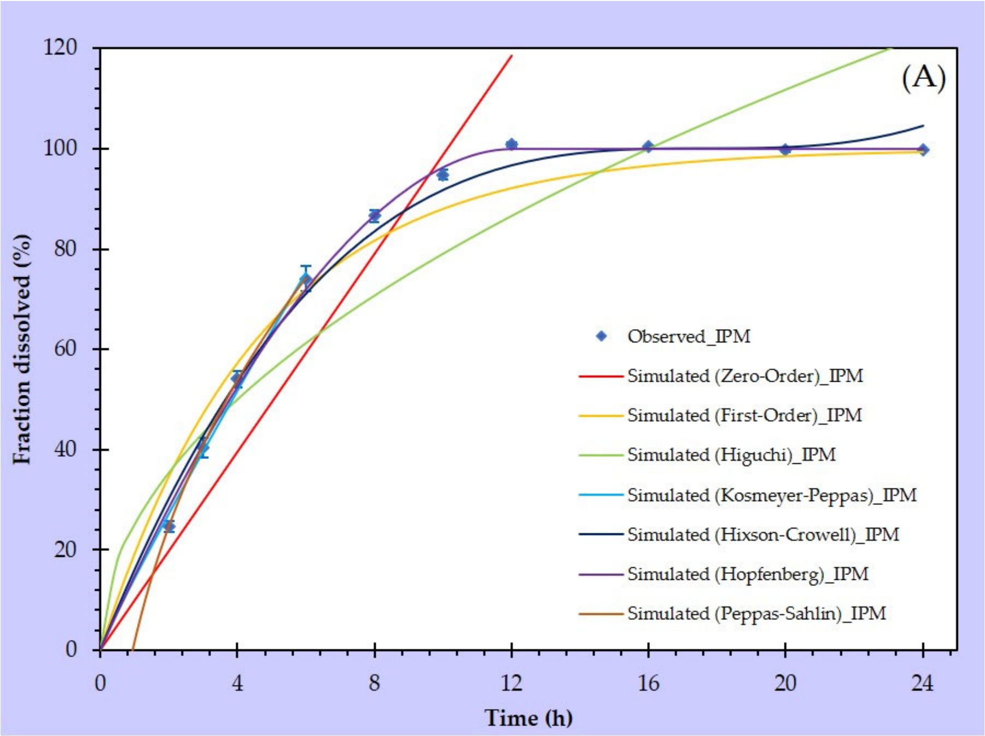

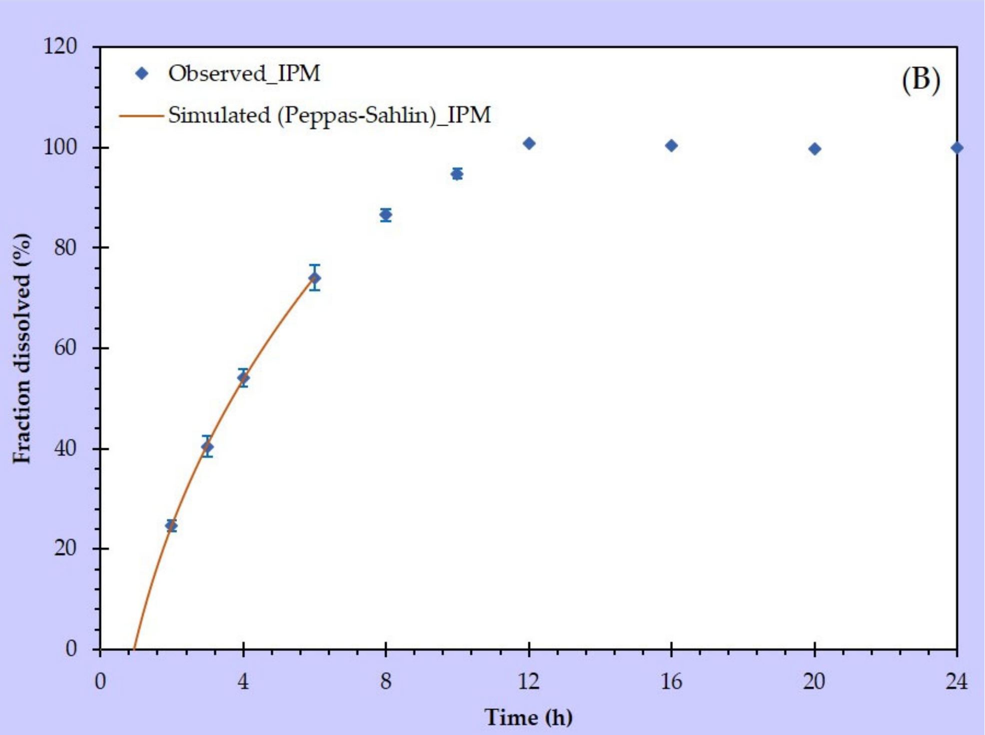

Figure S4. Mathematic model fitting of drug release profiles of IPM in phosphate buffer pH 6.8: (A) All mathematic models fitting, (B) Best mathematic model fitting

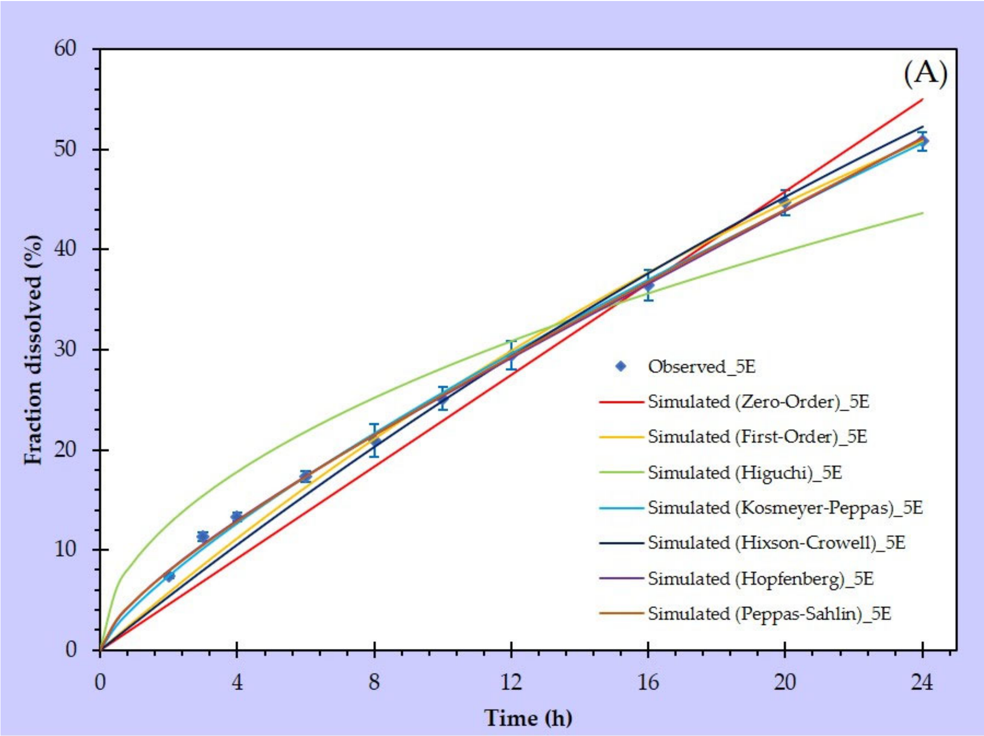

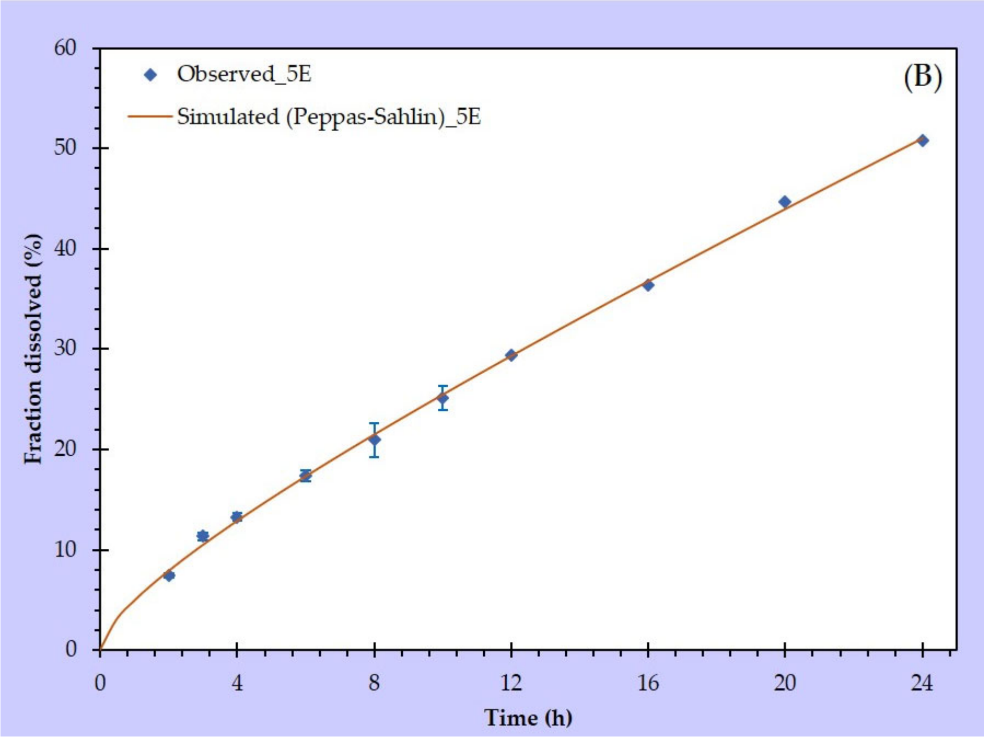

Figure S5. Mathematic model fitting of drug release profiles of 5E in phosphate buffer pH 6.8: (A) All mathematic models fitting, (B) Best mathematic model fitting

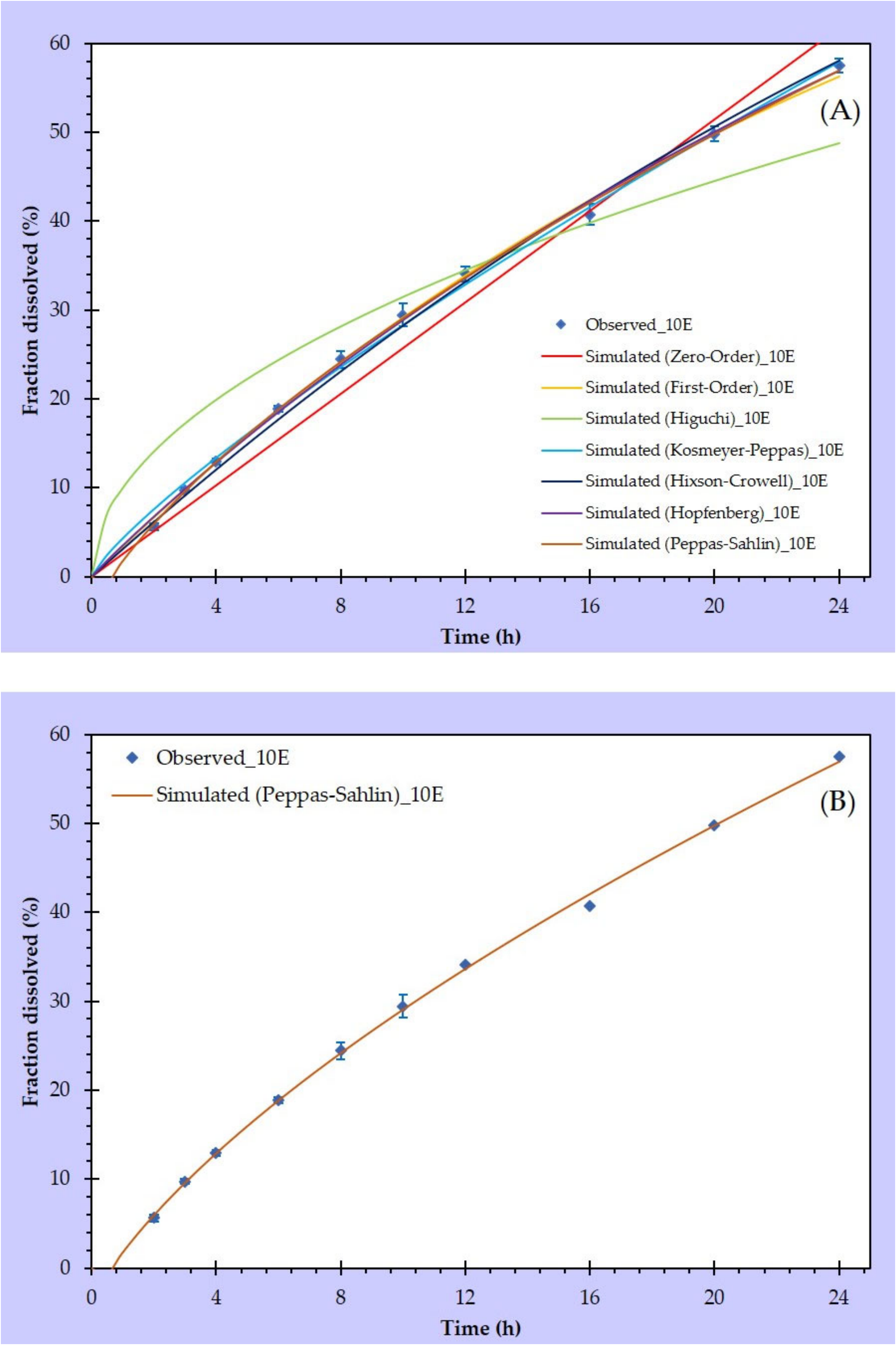

Figure S6. Mathematic model fitting of drug release profiles of 10E in phosphate buffer pH 6.8: (A) All mathematic models fitting, (B) Best mathematic model fitting

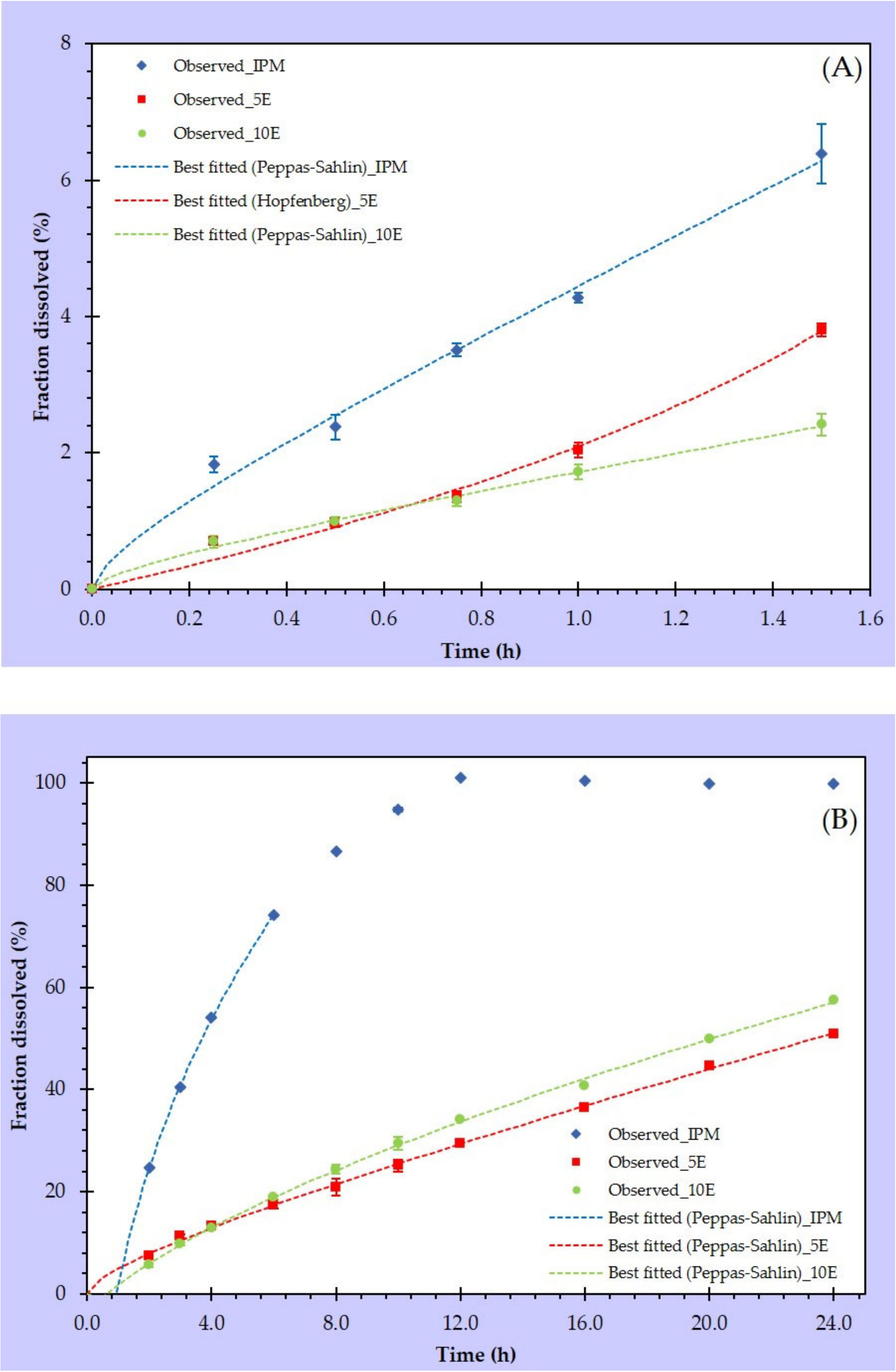

Figure S7. Best mathematic model fitting of drug release profiles of effervescent matrix tablets in (A) 0.1 N HCl buffer and (B) phosphate buffer pH 6.8
